# Supplementary material for: Repurposing Metabolic Inhibitors in the Treatment of Colon Adenocarcinoma Patient-Derived Models
Source: Cells. 2023 Dec 18;12(24):2859. doi: 10.3390/cells12242859 (PMC10742000; doi:10.3390/cells12242859)
Supplement: Supplementary file 1 [file cells-12-02859-s001.zip › cells-2754086-supplementary.pdf]

Supplementary Figure S1

A

Drug: 2DG (1) [mM]
Drug: Phenformin (2) [mM]
Drug Combo: 2DG+Phen (3) (1+2)

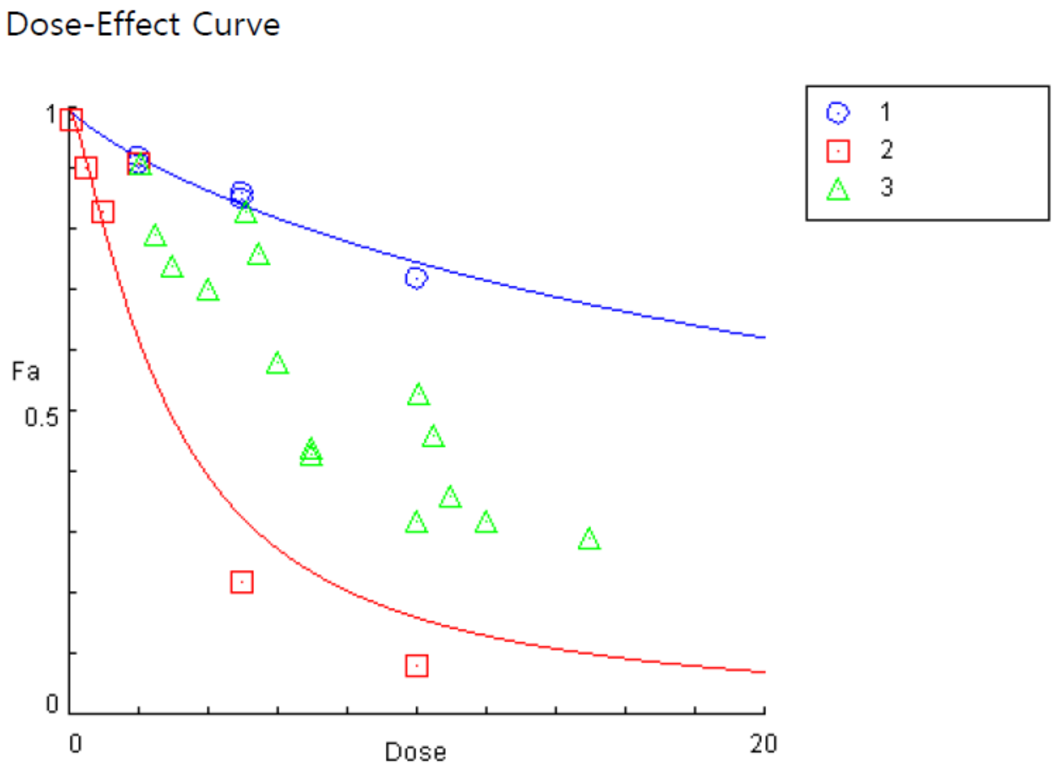

B

CI Data for Non-Constant Combo: 3 (1+2)

**Dose 1**	**Dose 2**	**Effect**	**CI**
2.0	0.1	0.91	1.07113
2.0	0.5	0.79	0.74024
2.0	1.0	0.74	0.95709
2.0	2.0	0.7	1.46838
2.0	5.0	0.44	1.49151
5.0	0.1	0.83	1.03135
5.0	0.5	0.76	0.96142
5.0	1.0	0.58	0.64705
5.0	2.0	0.43	0.66158
5.0	5.0	0.32	1.04212
10.0	0.1	0.53	0.35807
10.0	0.5	0.46	0.38321
10.0	1.0	0.36	0.36523
10.0	2.0	0.32	0.50725
10.0	5.0	0.29	0.98140

Supplementary Figure S1
CompuSyn analysis of cytotoxicity data was used to determine synergistic effect between 2DG and phenformin in HT29 cells. (A) Dose-effect plots of 2DG, phenformin, and 2DG + phenformin. (B) CI data from CompuSyn analysis present that the CI values of <1 indicated synergism between 2DG and phenformin.

Supplementary Figure S2

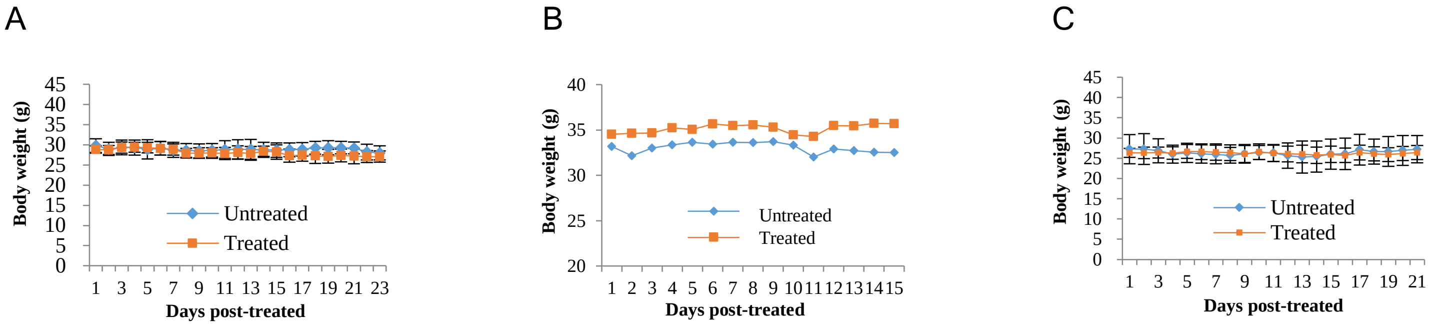

Supplementary Figure S2
Animal body weights were monitored during the experiment. (A) HT29 xenograft experiments, (B) PDX1 experiments, (C) PDX2 experiments.
